# Gemcitabine-Induced Radiation Recall Phenomenon in Cervical Cancer: A Case Report

**DOI:** 10.7759/cureus.39228

**Published:** 2023-05-19

**Authors:** Jesus Paula Carvalho, Daniela Freitas, Samir Hanna, Isabela A Velho, Filomena M Carvalho

**Affiliations:** 1 Obstetrics and Gynecology, Instituto do Cancer do Estado de Sao Paulo (ICESP/HC/FMUSP) Faculdade de Medicina. Universidade de Sao Paulo, Sao Paulo, BRA; 2 Clinical Oncology, Hospital Sirio Libanes, Sao Paulo, BRA; 3 Radiation Oncology, Hospital Sirio Libanes, Sao Paulo, BRA; 4 Pathology, Faculdade de Medicina. Universidade de Sao Paulo, Sao Paulo, BRA

**Keywords:** gynecologic malignancies, uterus, radiation recall reaction, radiotherapy, radiation, completion hysterectomy, radiation therapy, cervical cancer, radiation recall, gemcitabine

## Abstract

The radiation recall phenomenon is a rare, massive inflammatory reaction induced by some chemotherapeutic agents in previously irradiated areas. When it occurs in the pelvis it looks like a recurrence. Recognizing this phenomenon is paramount to avoiding unnecessary surgical intervention and complications. Symptoms manifest as dermatitis, mucositis, myositis, esophagitis, colitis, proctitis, and pneumonitis in areas within the irradiation field. Most patients respond to clinical treatment with corticosteroids. Here, we describe a 47-year-old patient with cervical carcinoma, FIGO stage IIB, submitted to external beam radiotherapy and concomitant chemotherapy with cisplatin (40 mg/m^2^ weekly), followed by intracavitary brachytherapy. One month after the end of radiotherapy and chemotherapy, the patient underwent laparoscopic completion hysterectomy plus bilateral salpingo-oophorectomy, followed by three cycles of cisplatin 50 mg/m^2^ D1 and gemcitabine 1,000 mg/m^2^ D1 and D8. Four months after the surgery, she presented with a suspicious mass in the vaginal dome that proved to be an exuberant inflammatory reaction that regressed after treatment with corticosteroids.

## Introduction

The radiation recall phenomenon (RRP) is the rare, localized inflammation of previously irradiated areas triggered by chemotherapeutic agents. Symptom onset typically occurs within days or weeks of the chemotherapeutic trigger but can also appear after years of medication [1]. It manifests as dermatitis, mucositis, myositis, esophagitis, colitis, proctitis, and pneumonitis in areas within the irradiation field. Among the agents that cause RRP are anthracyclines, capecitabine, taxanes, vinblastine, etoposide, methotrexate, trimetrexate, edatrexate, and gemcitabine [2]. Several multidrug combinations can trigger RRP, including herbal products, vaccines, and immune checkpoint inhibitors [3,4].

Since the 1990s gemcitabine, a nucleotide analog, has been recognized as an active drug in platinum-resistant gynecologic cancer [5]. It later showed potent radiosensitizing properties in preclinical and clinical trials [6]. A regimen of concurrent cisplatin with radiation followed by gemcitabine-cisplatin chemoradiation was proposed because it resulted in small but significant improvements in treating locally advanced and high-risk early-stage patients [7]. However, gemcitabine and cisplatin combined with conventional radiotherapy resulted in high toxicity in patients with cervical cancer [8].

The first RRP case attributed to gemcitabine was reported in 1999 in a patient diagnosed with transitional cell carcinoma of the bladder [2]. Since then, several dozen cases of RRP, in various anatomic locations, have been reported in the literature. Here, we present the case of a patient with cervical carcinoma treated by concomitant chemotherapy with cisplatin and radiation followed by gemcitabine, who developed a mass in the vaginal dome corresponding to RRP.

## Case presentation

**Figure 1:**
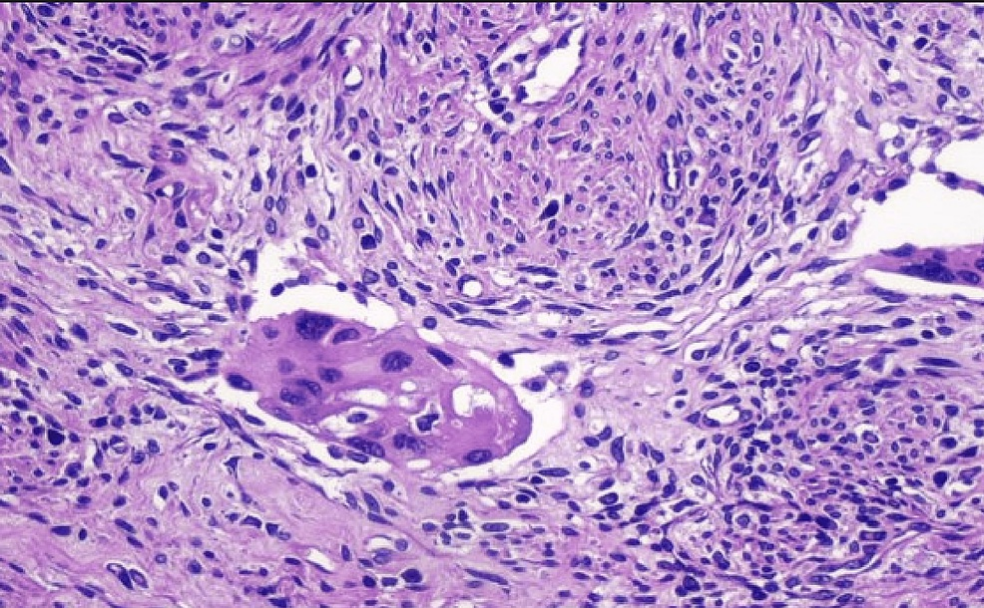
Pathological examination of the hysterectomy surgical specimen with minimal residual disease as isolated cells, and five cell clusters of up to 3 mm.

A 47-year-old female diagnosed with a bulky cervical adenocarcinoma, 6 cm size, stage IIB (FIGO-2018), underwent radiotherapy and concomitant chemotherapy with cisplatin 50 mg/m2 D1. The patient underwent external-beam radiotherapy using the IMRT + IGRT technique (intensity-modulated irradiation, and daily image guidance). The target volume of 50Gy in 25 fractions included the uterus, the parametria, and the upper one-third of the vagina. The target volume of 45Gy in 25 fractions included common, external, internal/obturator, and presacral iliac nodes. As for brachytherapy, the patient was given four insertions of Fletcher-type applicators, with a dose of 7 Gy being prescribed at point A in each of the insertions. Planning was done through tomography at each insertion. One month after the end of radiotherapy and chemotherapy, the patient underwent laparoscopic completion hysterectomy plus bilateral salpingo-oophorectomy. Because there was residual disease in the hysterectomy specimen (Figure 1), the patient was submitted to additional three cycles of cisplatin 50 mg/m^2^ D1 and gemcitabine 1,000 mg/m^2^ D1 and D8.

**Figure 2:**
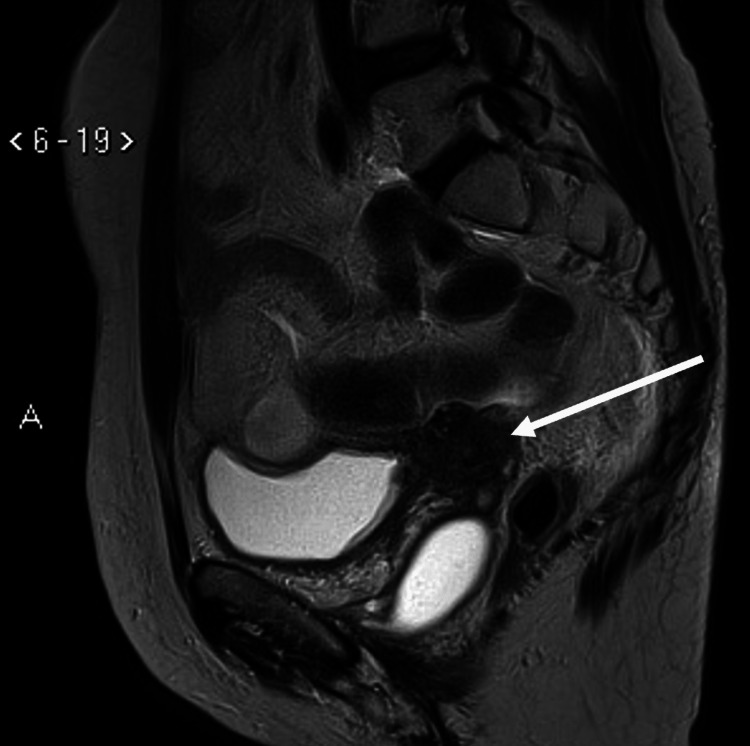
Magnetic resonance imaging demonstrated a solid mass measuring 6.3 x 2.2 x 3.0 cm, with a retractable aspect in the vaginal dome, presenting a low signal in T2 and enhanced by contrast.

Four months after surgery, the patient presented with bleeding in the vaginal dome. Gynecological examination showed a mass with a necrotic aspect suggestive of recurrence. Magnetic resonance imaging revealed a solid, retracted lesion measuring 6.2 x 2.2 x 3.0 cm adjacent to the vaginal dome. This lesion exhibited a low signal on T2, a medium signal on diffusion-weighted sequences, and late contrast enhancement on T1-weighted images. The lesion also extends bilaterally and involves the distal segments of both ureters, causing moderate upstream ureterohydronephrosis (Figure 2).

.

**Figure 3:**
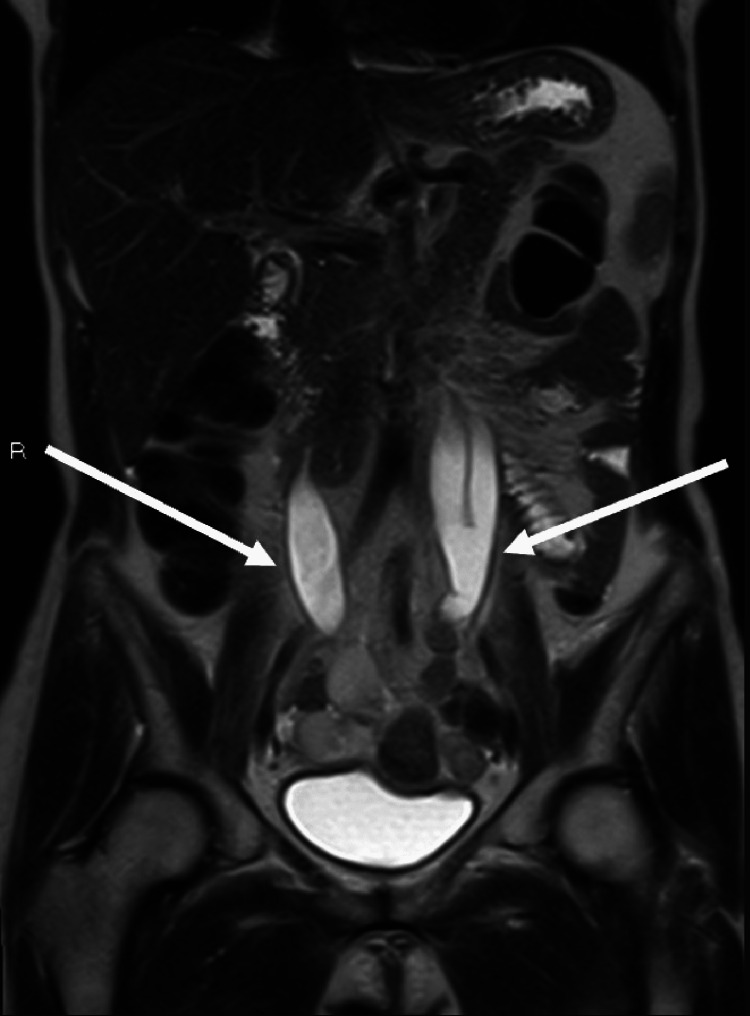
The mass presented lateral extension with apparent involvement and reduction in the caliber of the distal portion of both ureters, and moderate dilation of the collecting system above.

The mass presented lateral extension with apparent involvement and reduction in the caliber of the distal portion of both ureters, and moderate dilation of the collecting system above (Figure 3).

**Figure 4:**
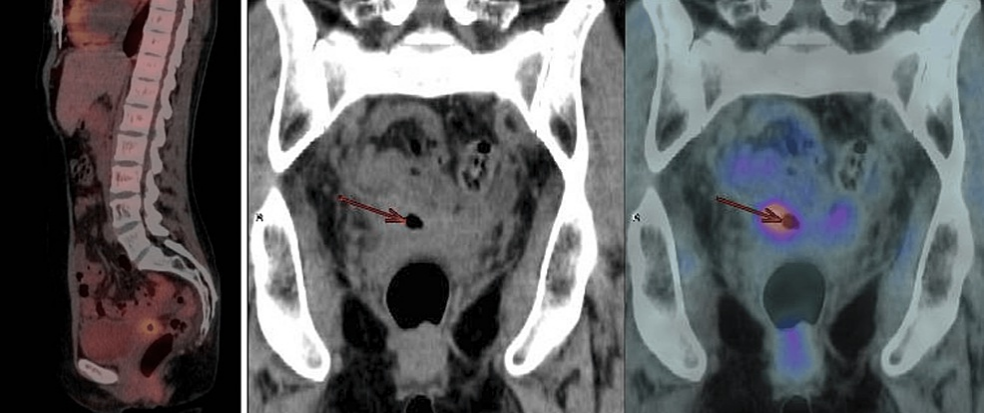
Positron emission tomography associated with computerized tomography demonstrated increased metabolic expression in the vaginal vault (SUVmax: 4.5 / late: 4.6). There was moderate dilatation of the bilateral ureters.

Positron emission tomography associated with computerized tomography demonstrated increased metabolic expression in the vaginal vault (SUVmax: 4.5 / late: 4.6). There was moderate dilatation of the bilateral ureters (Figure 4). 

**Figure 5:**
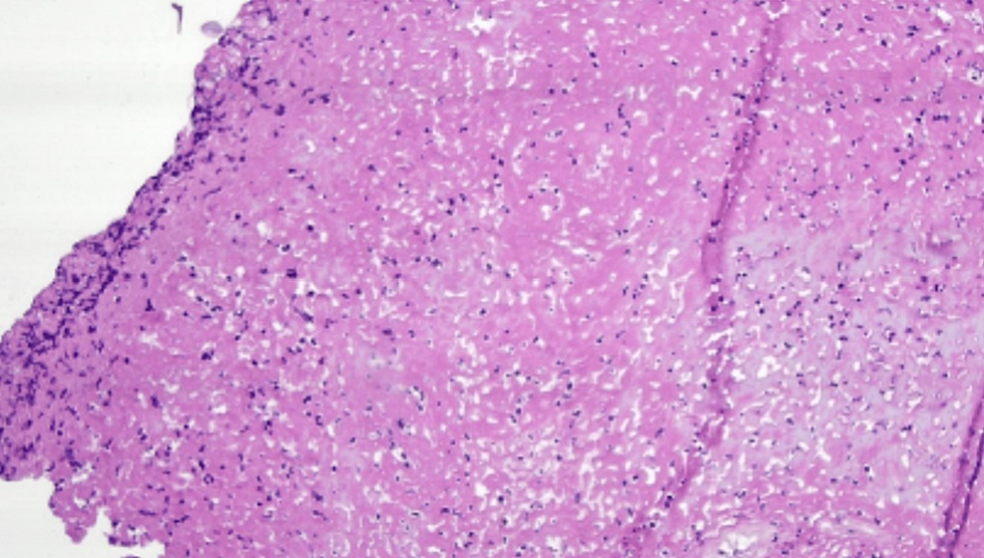
Biopsies of the mass on the vaginal dome showed necrotic tissue and no neoplastic cells.

Two consecutive biopsies of the mass on the vaginal dome showed necrotic tissue and no neoplastic cells (Figure 5).

**Figure 6:**
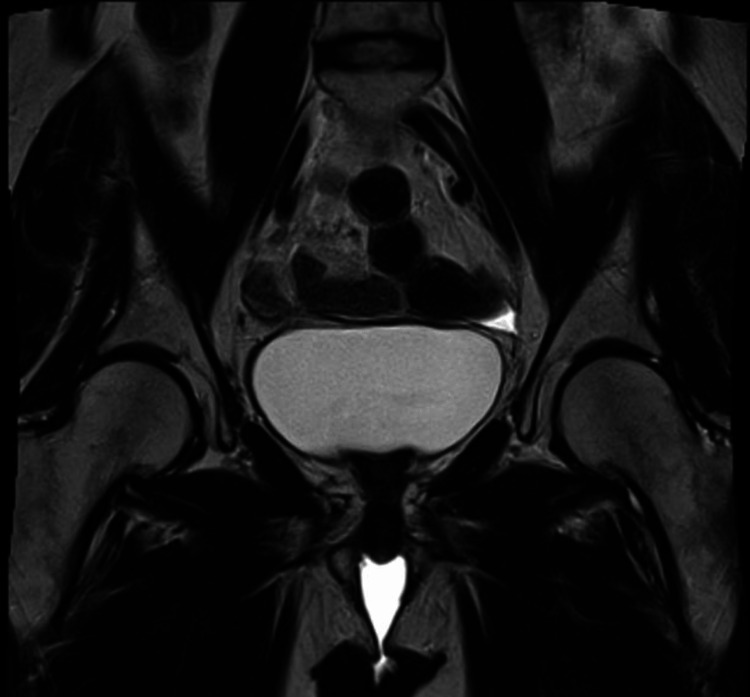
Magnetic resonance imaging showed only cicatricial changes next to the vaginal dome and minimal free liquid in the bottom of the peritoneal sac.

The patient was treated with dexamethasone 20 mg/day for four months. The vaginal lesion regressed, and bilateral hydronephrosis disappeared within four months. After five years of follow-up, the patient was asymptomatic without any disease evidence. Magnetic resonance imaging showed only cicatricial changes next to the vaginal dome and minimal free liquid in the bottom of the peritoneal sac, without lymph node enlargement (Figure 6).

## Discussion

RRP is an inflammatory reaction caused by some systemic agents when administered after radiotherapy. Dermatitis is the most common presentation. In a review conducted by Bhangoo et al., radiation recall dermatitis was reported in 129 cases (96 single-drug, 33 multi-drug) and was most frequently associated with docetaxel and gemcitabine [9]. Breast cancer was the most common tumor type in that study.

There are few reports of RRP in gynecological cancers. Schwartz et al. reported a 67-year-old patient with ovarian carcinoma who was treated with whole pelvic radiation followed by three courses of gemcitabine, and who developed RRP characterized by severe cellulitis [10]. Lemay et al. reported a young woman who had been treated for cervical cancer with radiation therapy and adjuvant gemcitabine and cisplatin chemotherapy. More than two months later she presented with a localized inflammatory process that was delimited by radiation fields and severe pain in the inguinal and upper abdominal regions, suggesting an RRP described as myositis [11]. Nishimoto et al. reported an 86-year-old woman with a history of radical hysterectomy and adjuvant radiotherapy for cervical carcinoma 36 years prior. She presented a second primary bladder squamous cell carcinoma and was treated by surgery and adjuvant systemic chemotherapy with gemcitabine and cisplatin. On day 13, the patient presented melena and multiple rectal ulceration, likely triggered by the administration of gemcitabine [12].

In phase 3, an open-label, randomized study, Dueñas-González et al. evaluated the addition of gemcitabine to concurrent cisplatin chemoradiotherapy for locally advanced cervical cancer. Two adjuvant 21-day cycles of cisplatin (50 mg/m^2^, on day 1) plus gemcitabine (1,000 mg/m^2^) were added, and the PFS at three years was significantly improved [13]. In the following years, several other studies incorporated gemcitabine in the treatment of advanced cervical cancer. Although gemcitabine plus cisplatin chemoradiotherapy followed by brachytherapy and adjuvant gemcitabine/cisplatin chemotherapy improved survival outcomes, toxicity was higher than with standard treatment [14]. However, RRP was not described as an adverse effect. 

Completion hysterectomy is not a standard of care but is an acceptable treatment for bulky cervical adenocarcinoma [15,16]. Factors associated with an increased risk of recurrence are histology, maximum tumor dimension, and tumor volume [17]. Our case was adenocarcinoma, 6 cm in size, and bulky tumor. Three phase 3 randomized trials have been published on the completion of surgery. They found a five-year DFS rate of 62% after hysterectomy compared to 53% without surgery for stage IB2 cervical cancer (P = 0.09) with a significant difference when comparisons were adjusted for tumor size, performance status, and age (P = 0.04) [16].

As far as we know, the present case is the first description of RRP resulting from gemcitabine concurrent to cisplatin chemoradiotherapy and completion hysterectomy, in locally advanced cervical cancer, mimicking a local recurrence. We will never know if surgical complication was potentialized by RRP or RRP potentialized by the surgery. It was an unexpected event for us. The original Dueñas-Gonzalez publication aroused great initial interest but with a significant increase in complications. [13] We considered it important to present this case precisely to warn about the risk of completion of hysterectomy in patients who received gemcitabine at any time.

## Conclusions

RRP is a rare massive inflammatory reaction that may occur even if radiotherapy was completed many years before. When it occurs in the pelvis it looks like a cancer recurrence and must be considered to avoid unnecessary surgical intervention. The treatment of choice is corticosteroids. We will never know if surgical complication was potentialized by RRP or RRP potentialized by the surgery. We considered it important to present this case report precisely to warn about the risk of completion of hysterectomy in patients who received gemcitabine at any time as part of the treatment.
